# Estimulação do Ramo Esquerdo: A Estimulação Cardíaca Mudou para Sempre?

**DOI:** 10.36660/abc.20211004

**Published:** 2022-02-14

**Authors:** José Carlos Pachón-M, Juán Carlos Pachón-M, Carlos Thiene C. Pachón

**Affiliations:** 1 Faculdade de Medicina Universidade de São Paulo São Paulo SP Brasil Faculdade de Medicina da Universidade de São Paulo, São Paulo, SP – Brasil; 2 Instituto Dante Pazzanese de Cardiologia São Paulo SP Brasil Instituto Dante Pazzanese de Cardiologia, São Paulo, SP – Brasil; 3 Hospital do Coração de São Paulo São Paulo SP Brasil Hospital do Coração de São Paulo, São Paulo, SP – Brasil

**Keywords:** Marca-passo Cardíaco Artificial, Ressincronização Cardíaca, Cardiomiopatia Dilatada, Bradiarritmias, Síncope

Sim, o velho sonho, a estimulação do sistema His-Purkinje, tornou-se realidade e agora está substituindo a estimulação convencional. Nesse sentido, o artigo “Estimulação do Ramo Esquerdo do Sistema His-Purkinje: Experiência Inicial” é um grande passo e deve ser lido não só pelo estimulista, mas também pelo cardiologista geral e pelo eletrofisiologista.^1^

Nas décadas de 60 e 70, o principal objetivo do marca-passo era corrigir a frequência cardíaca na síndrome de Stokes-Adams com bloqueio AV total. Os marca-passos eram dependentes de eletrodos epicárdicos, implantados por toracotomia. Entretanto, em 1959 Furman introduziu a estimulação endocárdica,^2^ sem toracotomia, tornando-se o padrão da estimulação cardíaca moderna na ponta do ventrículo direito. O sucesso da estimulação cardíaca foi extraordinário. No entanto, uma importante limitação surgiu devido à falta de sincronismo atrioventricular dando origem a um primeiro problema: a “Síndrome do marca-passo”,^3^ cuja prevenção deu origem ao marca-passo atrioventricular.

## Um efeito colateral oculto e deletério

Apesar do grande benefício da estimulação AV sincronizada, o eletrodo endocárdico no ápice do ventrículo direito promove uma estimulação indesejada^4^ com QRS largo e efeito de bloqueio completo do ramo esquerdo, com dessincronia significativa das paredes do ventrículo esquerdo dando origem a um segundo problema: a “Síndrome Ventricular do Marca-passo ou Síndrome do QRS Largo”,^7^ cuja prevenção deu origem ao ressincronizador.^5 - 8^ Apesar de estar presente em toda a estimulação cardíaca, essa dissincronia foi negligenciada por muito tempo devido ao grande benefício resultante da correção da bradicardia.

## Cardiomiopatia Induzida por Marca-passo (CMIMP)

A CMIMP é causada pela ativação do ventrículo esquerdo com QRS largo, condição que promove dessincronia miocárdica, disfunção sistólica e diastólica, dessincronia dos músculos papilares com insuficiência mitral, remodelamento ventricular e atrial, cardiomiopatia dilatada e aumento da mortalidade.^7 , 9^ Dentre vários critérios, podemos considerar CMIMP quando houver redução ≥10% da fração de ejeção sem outra causa que não a estimulação ventricular com QRS largo. Estima-se que ocorra em até 50% dos pacientes com marca-passo com ≥20% de estimulação ventricular por pelo menos 8 anos.^10^

## Como prevenir ou tratar a CMIMP?

Utilizando a “estimulação fisiológica” estimulando diretamente o feixe de His ou seu ramo esquerdo. Obviamente, a estimulação de qualquer músculo é muito mais eficaz se feita diretamente no nervo. Porém, infelizmente, o marca-passo convencional estimula diretamente o músculo (miocárdio), causando dessincronia. Os novos eletrodos de fixação ativa e de baixo perfil, manuseados com bainhas pré-moldadas, permitem estimular diretamente o “nervo” (o sistema de condução). Este recurso mudou definitivamente a estimulação cardíaca moderna.

## His-Bundle Pacing: novo padrão-ouro da estimulação cardíaca

Nos casos de bloqueio AV com sistema His-Purkinje preservado, a melhor estimulação possível é a estimulação do feixe de His. Não há nada que possa obter uma ressincronização melhor. Portanto, sua estimulação é o novo padrão-ouro na estimulação cardíaca moderna. Comparado com a estimulação convencional do ventrículo direito, mostra uma redução significativa no desfecho combinado de morte, hospitalização por insuficiência cardíaca ou upgrade para estimulação biventricular (FC=0,65, p=0,02 com estimulação ventricular >20%).^11^ No entanto, essa estimulação tem algumas desvantagens, como dificuldade técnica, limiares altos, onda R reduzida e detecção indesejável da onda P.

## Por que estimular o Ramo Esquerdo do Feixe de His?

Felizmente, a estimulação do ramo esquerdo do feixe de His, pela via interventricular transeptal, tem praticamente o mesmo resultado que a estimulação de His, mas sem os inconvenientes.^12^ Em 2005, buscando melhores opções de ressincronização cardíaca, propusemos à Medtronic um eletrodo de baixo perfil, com parafuso de fixação endocárdica longo, isolado eletricamente, porém com apenas 1mm eletricamente ativo na porção distal. O objetivo era penetrar profundamente no feixe de His ou no septo interventricular para estimular exclusivamente o sistema de condução His-Purkinje sem estimular o miocárdio circundante. Embora esse modelo ainda não tenha sido disponibilizado comercialmente, a Medtronic lançou um eletrodo similar não isolado, que se aproxima desse desenho, permitindo a penetração no septo interventricular e a estimulação do ramo esquerdo do feixe de His. Embora, a nosso ver, ainda não seja o ideal, os resultados são altamente promissores.^12^ O implante conta com uma bainha pré-moldada que facilita a penetração do eletrodo de forma perpendicular no septo interventricular, possibilitando a captura do ramo esquerdo.^1^ Isso permite uma ativação praticamente normal do ventrículo esquerdo, geralmente resultando em QRS de duração normal ou pelo menos com um tempo de ativação ventricular esquerda normal (LVAT ≤76ms).^13^

## Ainda há lugar para estimulação biventricular?

Sim, a ressincronização biventricular ainda é normalmente indicada quando a ressincronização não puder ser obtida usando o feixe de His ou a estimulação do ramo esquerdo. Atualmente, consideramos que, nos casos com indicação de ressincronização, de acordo com as diretrizes Deca/Sobrac, quando o LVAT ≤76ms não for alcançado com a estimulação do ramo esquerdo, deve ser realizada a estimulação biventricular entre o ramo esquerdo e o ventrículo esquerdo, com eletrodo adicional através do seio coronário e de uma veia cardíaca ou um eletrodo epicárdico ou intraventricular esquerdo. Essa abordagem certamente será incorporada às diretrizes nos próximos anos.
