# Supplementary material for: Estimulação do Ramo Esquerdo: A Estimulação Cardíaca Mudou para Sempre?
Source: Arq Bras Cardiol. 2022 Feb 14;118(2):517–8. [Article in Portuguese] doi: 10.36660/abc.20211004 (PMC8856693; doi:10.36660/abc.20211004)
Supplement: Translation

# Translation: Left Bundle Pacing: Has Cardiac Pacing Changed Forever?

**Authors:** José Carlos Pachón-M, Juán Carlos Pachón-M, Carlos Thiene C. Pachón

**Affiliations:** 1 Brazil Faculdade de Medicina da Universidade de São Paulo, São Paulo, SP – Brazil; 2 Brazil Instituto Dante Pazzanese de Cardiologia, São Paulo, SP – Brazil; 3 Brazil Hospital do Coração de São Paulo, São Paulo, SP – Brazil

**Keywords:** Cardiac Pacemaker Artificial, Cardiac Resynchronization, Dilated Cardiomyopathy, Bradyarrhythmias, Syncope

Yes, the old dream, the stimulation of the His-Purkinje system, has become reality and is now replacing the conventional pacing. In this sense, the article “Stimulation of the Left Branch of the His-Purkinje System: Initial Experience” is a great step and should be read not only by the stimulist, but also by the general cardiologist and by the electrophysiologist.^1^

In the 60s and 70s, the main purpose of the pacemaker was to correct the heart rate in Stokes-Adams syndrome with total AV block. Pacemakers were dependent on epicardial leads, implanted by thoracotomy. However, in 1959 Furman introduced the endocardial pacing,^2^ without thoracotomy, becoming the standard of modern cardiac pacing in the right ventricular apex. The success of the cardiac stimulation was extraordinary. Nevertheless, an important limitation emerged due to the lack of atrioventricular synchronism given origin to a first problem: The “Pacemaker Syndrome”,^3^ whose prevention gave rise to the atrioventricular pacemaker.

## A hidden and deleterious side effect

Despite the great benefit of the AV synchronized pacing, the endocardial lead at the apex of the right ventricle remained causing unwanted pacing^4^ with wide QRS and complete left bundle branch block effect, with significant dyssynchrony of the left ventricular walls given rise to a second problem: the “Ventricular Pacemaker Syndrome or Wide QRS Syndrome”,^7^ whose prevention and treatment gave origin to resynchronization therapy.^5 - 8^ . Despite being present in the entire cardiac stimulation, this dyssynchrony was neglected for a long time due to the great benefit resulting of the bradycardia correction.

## Pacemaker Induced Cardiomyopathy (PICM)

PICM is caused by the activation of the left ventricle with a wide QRS that promotes myocardial dyssynchrony, systolic and diastolic dysfunction, papillary muscle dyssynchrony with mitral regurgitation, ventricular and atrial remodeling, dilated cardiomyopathy, and increased mortality.^7 , 9^ Among several criteria, we may consider PICM at least when there is ≥10% reduction in ejection fraction with no other cause than the ventricular pacing with a wide QRS. It is estimated to occur in up to 50% of pacemaker patients with ≥20% of ventricular stimulation for at least 8 years.^10^

## How to prevent or treat PICM?

By “physiological pacing” stimulating directly the His bundle or its left branch. Obviously, stimulation of any muscle is much more effective if done directly on the nerve. However, unfortunately, the conventional pacemaker stimulates the muscle (myocardium), causing dyssynchrony. The new active fixation and low-profile leads, handled with pre-shaped sheaths, allow one to stimulate directly the nerve (the conduction system) and have definitely changed the modern cardiac pacing.

## His-Bundle Pacing: New Gold-Standard of Cardiac Pacing

In cases with AV block with preserved His-Purkinje system, the best possible stimulation is the His bundle pacing. There is nothing that can get better resynchronization. Therefore, His pacing is the new gold-standard in modern cardiac pacing. Compared with right ventricular conventional pacing, it shows a significant reduction in the combined endpoint of death, heart failure hospitalization, or upgrade to biventricular pacing (HR=0.65, p=0.02 with ventricular pacing >20%).^11^ However, this stimulation has some pitfalls, such as technical difficulty, high thresholds, reduced R wave, and undesirable P wave detection.

## Why stimulate the Left Branch of the His Bundle?

Fortunately, stimulation of the left branch of the His bundle, via the transseptal interventricular route, has practically the same result as the His stimulation, but without the drawbacks.^12^ In 2005, searching for better cardiac resynchronization options, we proposed to Medtronic a low-profile lead, with a longer isolated screw, but with only 1mm electrically active in the distal portion. The aim was to penetrate deep into the His bundle or into the interventricular septum to exclusively stimulate the His-Purkinje conduction system without stimulating the surrounding myocardium. Although this model has not yet been made commercially available, Medtronic has launched a similar non-isolated lead, which approximates this design, allowing penetration into the interventricular septum and stimulation of the left branch of the His bundle. Although, in our view, it is still not ideal, the results are highly promising.^12^ The implant relies on a pre-shaped sheath that facilitates the perpendicular penetration into the interventricular septum, making it possible to capture the left bundle branch.^1^ This allows for an almost normal activation of the left ventricle, commonly resulting in QRS of normal duration or at least a normal left ventricular activation time (LVAT ≤76ms).^13^

## Is there still a place for Biventricular Pacing?

Yes, biventricular resynchronization is still normally indicated when resynchronization cannot be achieved using His bundle or left bundle branch pacing. Currently, we consider that, in cases with indication for resynchronization, according to Deca/Sobrac guidelines, when LVAT ≤76ms is not reached with left bundle pacing, biventricular stimulation between the left bundle and the left ventricle must be performed, with an additional lead through the coronary sinus and cardiac vein, or even epicardial or intraventricular lead. This approach will certainly be incorporated into the guidelines in the coming years.
